# Supplementary material for: How to measure bacterial genome plasticity? A novel index helps gather insights on pathogens
Source: Microb Genom. 2025 Aug 4;11(8):001459. doi: 10.1099/mgen.0.001459 (PMC12321203; doi:10.1099/mgen.0.001459)
Supplement: Uncited Supplementary Material 1. [file mgen-11-01459-s001.pdf]

Supplementary Material

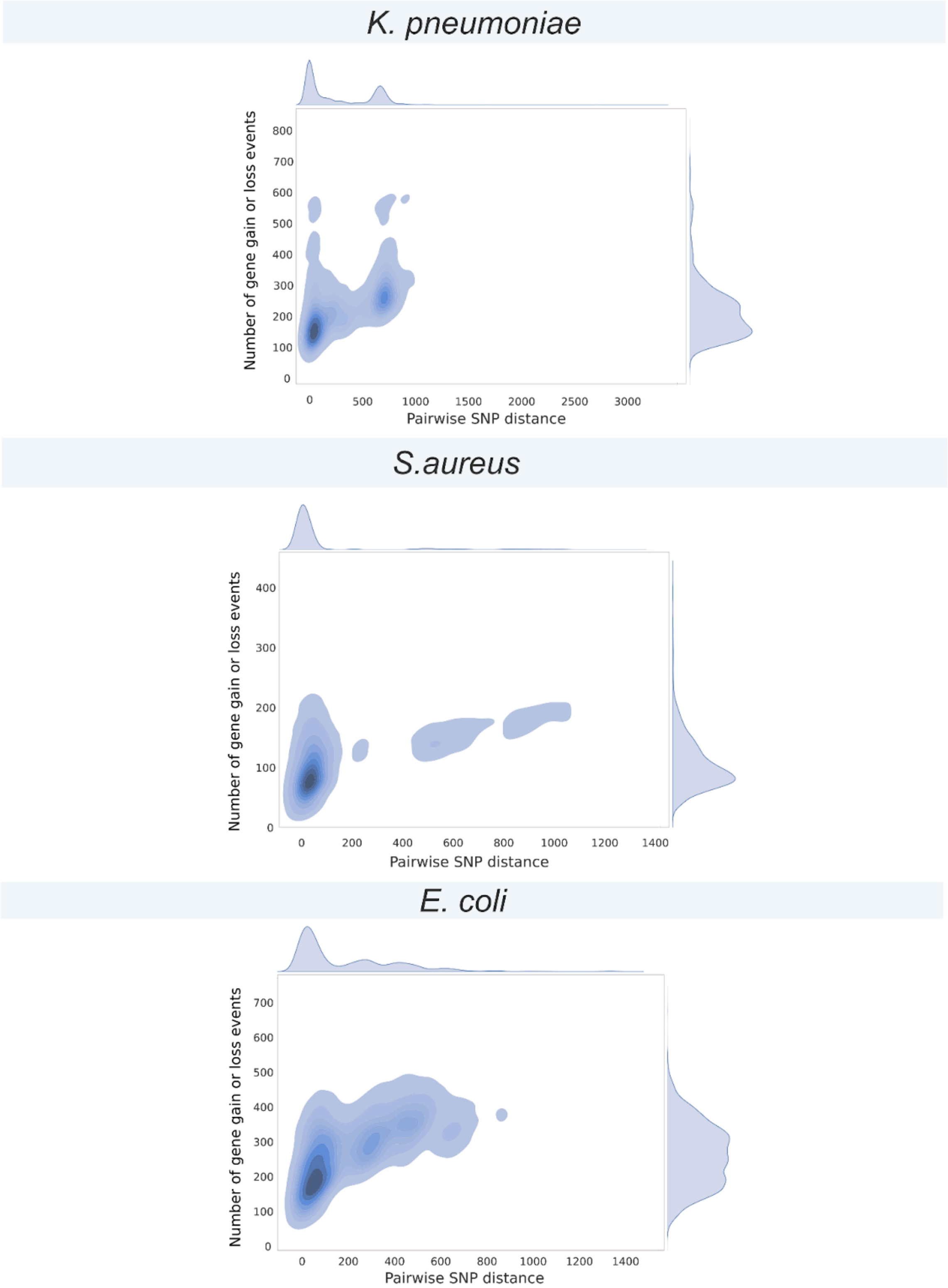

Supplementary Figure 1. Density plots of Number of gene gain or loss events against the SNP distance for all pairs within each fastBAPS cluster for *K. pneumoniae*, *S. aureus* and *E. coli*.

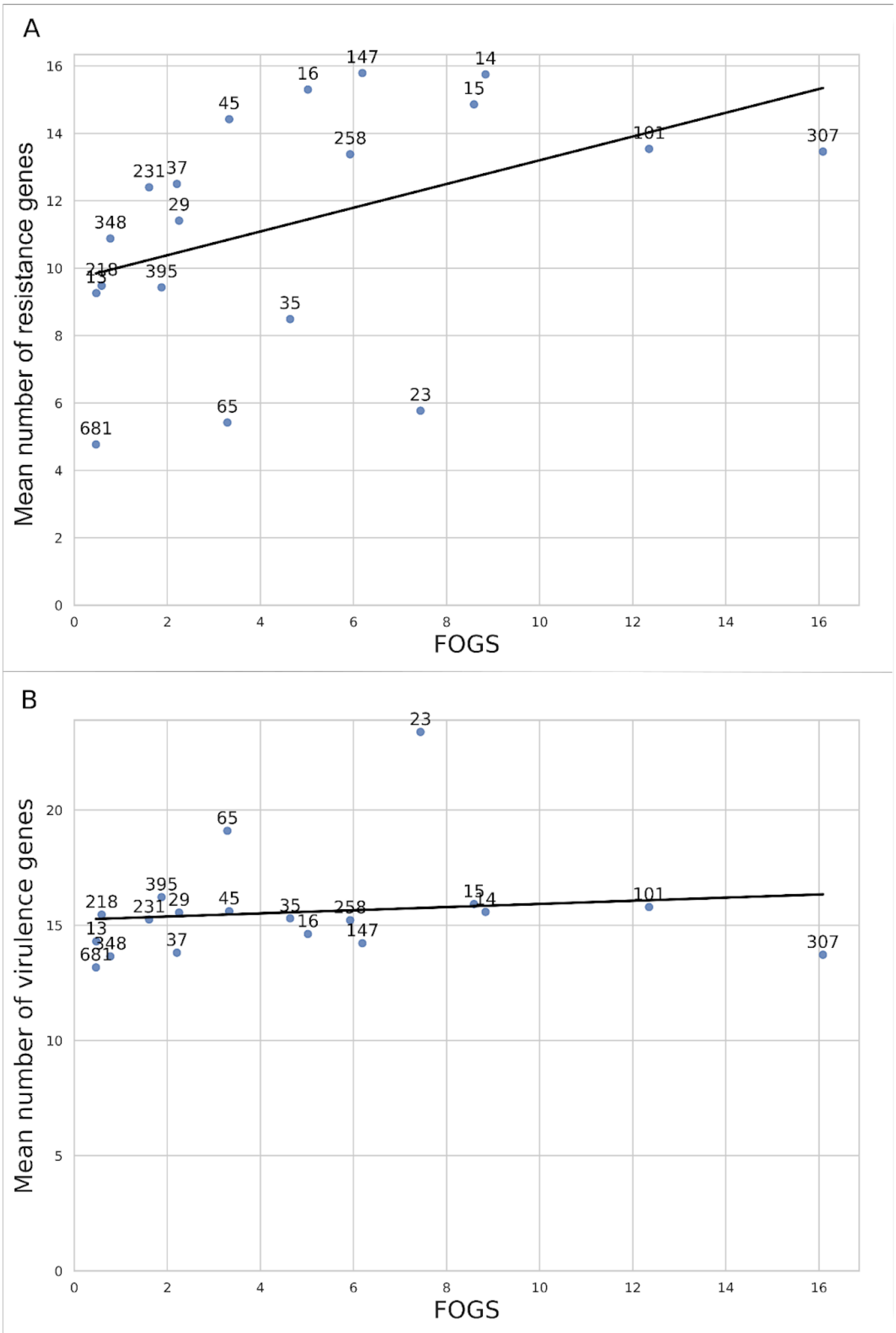

Supplementary Figure 2. A) Mean number of resistance genes against FOGS within each *K. pneumoniae* cluster. The regression line is presented in gray (R^2=0.43 , p-value<0.05). B) Mean number of virulence functions (VFDB) against FOGS within each *K. pneumoniae* cluster. The regression line is presented in gray (R^2=0.12 , p-value>0.05).

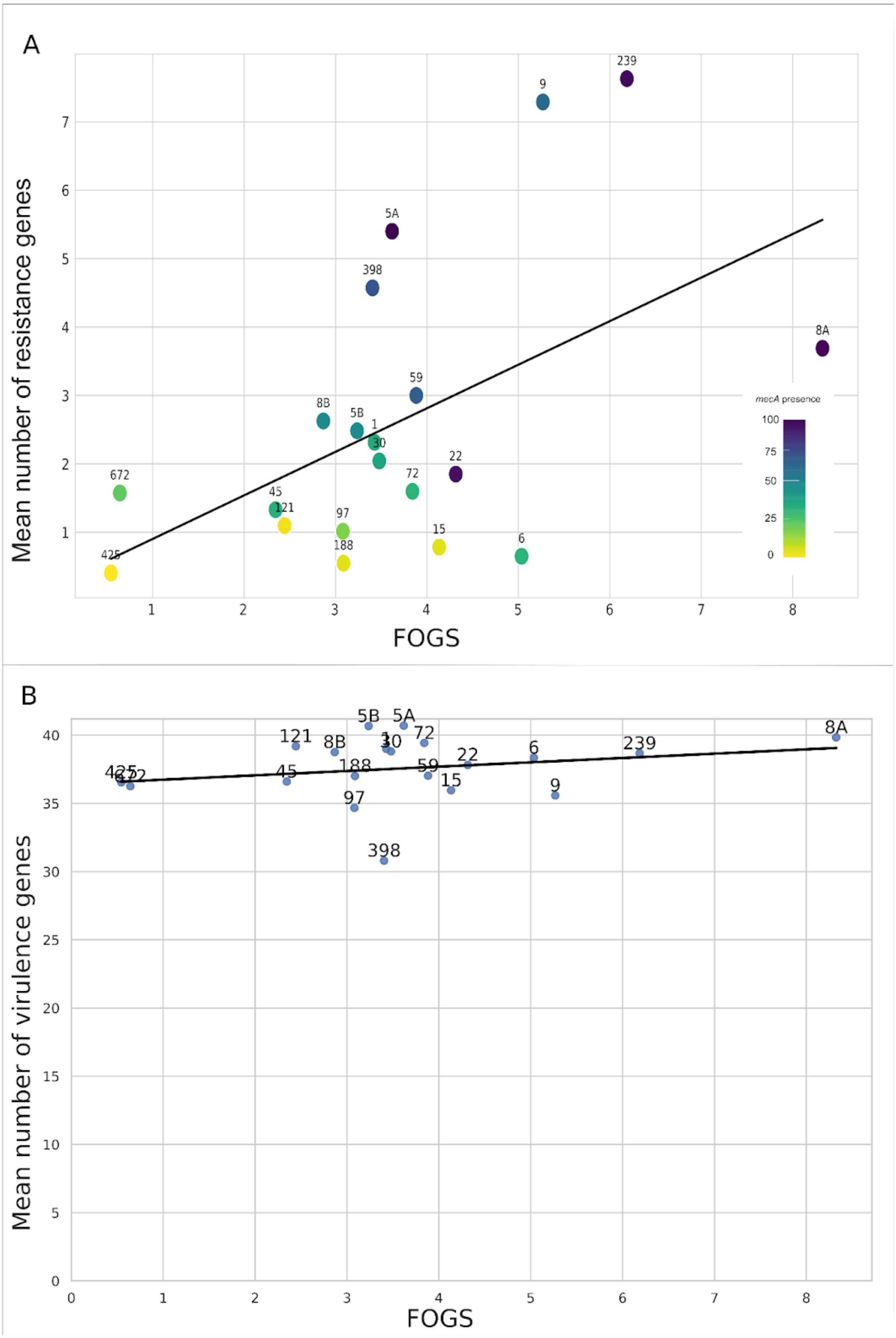

Supplementary Figure 3. A) Relationship between FOGS and the mean number of resistance genes in *Staphylococcus aureus*. The color of the point indicates the prevalence of the *mecA* within each cluster.B) Relationship between FOGS and the mean number of virulence genes.
